# Does the practice of blood film microscopy for detection and quantification of malaria parasites in northwest Ethiopia fit the standard?

**DOI:** 10.1186/s12913-014-0529-x

**Published:** 2014-11-01

**Authors:** Fantahun Biadglegne, Yeshambel Belyhun, Jemal Ali, Fisha Walle, Nigussu Gudeta, Afework Kassu, Andargachew Mulu

**Affiliations:** Faculty of Medicine and Health Sciences, Bahir Dar University, Bahir Dar, Ethiopia; School of Biomedical and Laboratory Science, College of Medicine and Health Sciences, University of Gondar, Gondar, Ethiopia; Amhara Regional State Health Research Laboratory, Bahir Dar, Ethiopia; Management Sciences for Health, Amhara Regional State, Bahir Dar, Ethiopia; Institute of Medical Microbiology and Epidemiology of Infectious Diseases, University of Leipzig, Leipzig, Germany; Institute of Virology, Medical Faculty, University of Leipzig, Leipzig, Germany

**Keywords:** Health service, Laboratory service, Northwest Ethiopia

## Abstract

**Background:**

The diagnosis of malaria in clinical laboratories mainly depends on blood smear microscopy and this technique remains the most widely used in Ethiopia. Despite the importance of blood smear microscopy for patient’s diagnosis and treatment, little effort has been made to precisely determine and identify sources of error in malaria smear microscopic diagnosis and quantification of parasitaemia. The main objective of the present study was to assess the laboratory practices of health care laboratories carrying out blood films microscopy.

**Methods:**

A cross sectional study was conducted in northwestern Ethiopia involving 29 health care institutes. A structured and pretested questionnaire were used to collect relevant information on the physical conditions, laboratory logistics and laboratory practices carrying out blood smear microscopy.

**Results:**

There was inadequacy of laboratory reagents, guidelines and materials. Most of the health institutes have been practicing re-utilization of microscope slides for malaria microscopy. The technical procedure (preparing of reagents, making of blood films and staining of the slides) were found to be below the standard in 50% of the health institutes. Refresher training and quality assessment has been done only in two and six of the health institutes in the past five years, respectively.

**Conclusion:**

In most of the health care laboratories studied, availability of laboratory logistics and technical practices for malaria microscopy were found to be below the standard set by World Health Organization. Improving logistics access for malaria microscopy at all level of health care is important to increase accuracy of diagnosis and quantification of malaria parasites. Moreover, continued training and regular supervision of the staff and implementation of quality control program in the area is also crucial.

## Background

Malaria remains one of the major causes of morbidity and mortality in developing world. According to World Health Organization (WHO) report in 2013 it has been estimated that 207 million cases and 627,000 deaths occurred globally in 2012 [1]. And about 80% cases and 90% deaths were from Africa [1]. In Ethiopia, approximately 68% of the total population lives in areas at risk of malaria. Malaria is ranked as the leading communicable disease in Ethiopia, accounting for about 30% of the overall disability adjusted life years lost. It has been consistently reported as one of the three leading cause of morbidity and mortality in the past years [2]. In a non-endemic year, 5-6 million clinical malaria cases and over half million confirmed cases are reported from health facilities [3]. As the potential health service coverage is minimum and due to the low health service utilization rate, the number of malaria cases reported by health facilities is only a portion of the actual magnitude [3]. However, the prevalence of malaria currently is remarkably declining. In Ethiopia *Plasmodium falciparum* and *Plasmodium vivax* are the predominant species [3,4].

Blood smear microscopy has been used to detect malaria parasites in the blood of infected patients since Laveran first identified the parasites in 1880 [5]. Microscopic examination of blood is the most affordable, accessible, widely used and reliable technique for diagnosis of malaria infection. Although molecular techniques for quantifying parasites have made significant progress in recent years, microscopy remains the primary technique for quantification of parasites. Microscopy is routinely relied upon as a primary endpoint measurement for epidemiological studies, intervention studies, and clinical practices [6]. Like any other developing countries of the tropical settings, the diagnosis of malaria in clinical laboratories in Ethiopia is depend almost exclusively on blood smear microscopy [7,8] and currently this technique is also widely used in parallel to rapid malaria test which become commonly available with an introduction of extension health workers in Ethiopia. Despite the importance of microscopy for patient’s diagnosis and treatment, little effort has been made to precisely determine and identify sources of error in microscopic diagnosis and quantification of parasitaemia and to secure quality control program for malaria microscopy. The Amhara Regional Health Bureau in general and the Amhara Regional Health Research laboratory in particular are responsible for the training and supervision of malaria microscopists and quality control program of malaria microscopy in the northwest Ethiopia. However, information on the existing laboratory conditions and procedures related to blood films microscopy for malaria parasites in Ethiopia is very scarce. The purpose of this study was therefore to asses the physical conditions and laboratory practices of health care laboratories carrying out blood film microscopy for diagnosis of malaria parasites in Amhara Regional State, northwest Ethiopia.

## Methods

A cross sectional health institutions based study was conducted in 29 health care laboratories undergoing blood smear microscopy for identification of malaria parasites in Amhara Regional State of northwest Ethiopia. Medical laboratory technician working in 29 health institutes were invited to attend a refresher course in the diagnosis of malaria and associated quality control and management programs. The workshop was organized by Malaria and other Vector Borne Disease Department, Amhara Regional State Health Bureau, Amhara Regional Health Research Laboratory and Department of Microbiology and Parasitology, College of Medicine and Health Sciences of the University of Gondar.

A self administered, structured and pretested questionnaire was used to collect the following information: physical conditions of laboratories; the availability of stains, reagents and guidelines for reagent preparations; the availability, types and quantity of microscopes and microscope slides; availability of atlas. Data on technical issues of reagents preparation, smearing, staining, reading of the microscope slides, reporting pattern of the findings and recoding and documentation were also collected and analyzed as per the standard set by WHO. Smeared slides read by the study participants were rechecked for the quality as well as for their correct identification of the parasite species by experienced microscopists who were blind for the status of respective slides. Data was entered and analyzed according to the set variables using SPSS 13 statistical soft ware.

The study was ethically approved by the Amhara Regional Health Research Laboratory which controls health care laboratories in the region and University of Gondar, Ethiopia. Moreover, written informed consent was obtained from each participant.

## Results

A total of 29 medical laboratory technicians with a mean age of 27.4 years (range 20-50 years) were included in the study. Of all, female participants were only 3 (10.3%). All of the respondents were qualified medical laboratory technicians who have got their studies in recognized training institutions in the country. The average work experience of the technicians was 4 years with a minimum of 6 months and a maximum of 22 years of service as technicians (Table 1). All of the laboratories included in the study were staffed by two technicians and lack laboratory clerks/aids. The status of laboratory equipments regarding blood smear microscopy is presented in Table 2. Out of the total health care laboratories included in the study, 14 (48.3%), 13 (44.8%) and 2 (6.9) had two, one and three microscopes, respectively. The type of the microscope varied and ranged from monocular with solar light to binocular with in-built lamp (Table 2).

**Table 1:** **Sociodemographic characteristics of study subjects in Amhara Regional State, Ethiopia, 2006**

**Characteristics**	**N (%)**
***Age in years***	
Mean age (range)	27.4 (20-50)
20-30	22 (75.9)
31-40	4 (13.8)
41-50	3 (10.3)
Total	29 (100)
***Sex***	
Male	26 (89.7)
Female	3 (10.3)
Total	29 (100)
***Educational level***	
Junior technicians	5 (17.2)
Senior technicians	6 (20.7)
Diploma	12 (41.4)
B. Sc	1 (3.4)
Malaria technician	5 (17.2)
Total	29 (100)
***Experience in practice (in years)***	
Mean (range)	4 (0.5-24)
<5	24 (82.9)
6-10	1 (3.4)
11-15	1 (3.4)
>16	3 (10.3)
Total	29 (100)
***Working area (Health Institutes)***	
Referral Hospital	1 (3.4)
Rural Hospital	2 (6.9)
Health Center	22 (75.9)
Clinics	4 (13.5)
Total	29 (100)

**Table 2:** **Status of health care laboratories on basic equipments/reagents for carrying out blood smear microscopy for diagnosis of malaria parasite in Amhara Regional State, Ethiopia, 2006**

**Lab equipments/Reagents**	**No (%) of laboratories**
***No of microscope***	
One	13 (44.8)
Two	14 (48.3)
Three	2 (6.9)
***Type of microscope***	
Binocular with natural solar light	21 (72.4)
Binocular with external light	1 (3.6)
Binocular with inbuilt lamp	4 (14.3)
Monocular with natural solar light	3 (10.3)
Monocular with external light	0 (0)
Monocular with inbuilt lamp	0 (0)
***Kinds of stains***	
Giemsa	21 (72.4)
Wright	0 (0)
Both	6 (20,7)
None	2 (6.9)
**Guide line for reagent preparation**	
Present	5 (17.2)
Absent	24 (82.8%)
Adequate number of slides	16 (55.2)
Drying rack	21 (72.4)

In 21 (72.4%) of the laboratories, Giemsa staining solution is available in the form of stock solutions. However, in about 24 (82.8%) of the laboratories, there was no guideline for reagent preparation and staining. A quarter of the facilities with Giemsa stain had difficulties in getting the stain. In more than half of the health care laboratories with scarcity of microscopic slides, laboratory technician used to practice reutilization of slides. Of the total laboratory workers included in the study, 15 (52%) of them reported that, they had not participated in epidemic investigation and/or control program in their service years (Table 3). Although all of the respondents knew very well about the importance of refresher training, only three of the technicians had got a chance to attend refresher training on malaria during their work experience.

**Table 3:** **Current practice in laboratories for identification of malaria parasite in Amhara Regional State, Ethiopia, 2006 (N = 29)**

**Variables**	**Frequency**
Availability of staining reagent	
Yes	23 (79.3)
No	6 (20.7)
Utilization of new slides for each sample	
Yes	5 (17.2)
No	24 (82.8)
Participation in epidemic investigation and/or control program	
Yes	14 (48.0)
No	15 (52.0)
Previous participation on refresher training on malaria microscopy	
Yes	3 (10.3)
No	26 (89.7)
Blood smear used	
Thick	7 (24.1)
Thin	8 (27.6)
Both	14 (48.0)
Quality control program done so far regarding blood smear	
Yes	6 (20.7)
No	23 (79.3)
Do you report species, stages and parasite density?	
Yes	28 (96.6)
No	1 (3.4)
Methods of parasite quantification	
Plus system of quantitation	27 (93.1)
Counting parasites/μl	2 (6.9)
Mean (range) number of blood films examined/day	
Per institution	46.5 (7-175)
Per individuals	23.5 (1-87)
Number of positives/day	
Mean (range)	18.8 (0-120)
0-40	22 (75.9)
41-80	5 (17.2)
≥80	2 (6.9)
Time spent to read a single blood film (in minutes)	
Mean (range)	10.8 (2-45)
2-20	17 (58.6)
21-39	10 (34.5)
≥40	2 (6.9)

All laboratories routinely examined blood films for identification and quantification of malaria parasites. Of these 7 (24%), 8 (27.6) and 14 (48%) of the technicians prepare thick blood smears, thin blood films and both thick and thin blood smears, respectively for identification of malaria parasites (Table 3). All of them responded that, they used registration book for recording and documenting of results. Despite the high rates of malaria parasites in the area and high work burden among technicians, in about 23 (79.3%) of the health institutes, there was no previous quality control program under taken by the responsible body. Out of the total participants, 96.6% of the respondents had experience on how to report on the species, stages and the parasite density, though nearly 50% of the respondent utilized both thick and thin blood films for the identification and quantification of malaria parasites. Most of them used abbreviations during reporting which do have a common understanding with other health care workers in their respective institutes, and the method of parasite quantification they used was plus system. The mean number of blood films examined per day in the institution and per individuals was 46.5 (with the minimum of 7 and maximum of 175) and 23.5 (with the minimum of 1 and maximum of 87), respectively. However, the mean number of positives samples per day was 18.8 with the minimum of zero and maximum of 120 during the malaria season. The average time spent to read a single blood smear slide was 10.8 minute with the minimum of 2 and maximum of 45 minutes. The time and quality of the smear done during the refresher training was in line with what they responded to the questionnaire checklist particularly 25 (85.2%) of them clearly identified the parasite species. However, the rest showed dilemma because of poor slide preparation although they identified the parasite species.

## Discussion

Clinical laboratory services are essential components of curative and preventive health care activities worldwide. Laboratory investigations are a vital part of the clinical assessment and laboratory results guide the selection of drugs for patient management [9]. In many developing countries including Ethiopia, however, laboratory services have been neglected due to chronic under-investment and professional biases by other health care workers [10-13]. One main element of malaria control strategy is effective case management by early detection and treatment of the patients [3], which would obliviously, be economically beneficial since many African countries including Ethiopia used expensive drugs as first line anti-malaria treatment [6,7]. This study indicates that in almost all of the health institutions included in the study, there were shortage of trained human power in the area, despite the high work load for a technician reached to read as many as 87 blood film slides far above what is recommended for one technician and the ever increasing demand from the patients and other health care providers which is in agreement with previous studies [10,12]. The need for continuous laboratory technician and/or local microscopists training for filling this gap at each health facility could bring a paramount importance to alleviate the problem. To make both microscopy and the use of rapid diagnostic tests (RDTs) especially for the uncomplicated malaria cases would be also the best possible solution although it is true that Ethiopia does not recommend using RDTs at health center and hospital levels. However, the policy ‘microscopy at health facility level and RDTs at community level’ has to be changed and both techniques should be available at the same time and useful whenever necessary.

The lack of guidelines and standard operational procedures in 82% and lack of adequate number of microscope slides (45%) of is in accordance with other previous studies [10-13] reported elsewhere in Ethiopia. These would lead to poor and inefficient laboratory practice and has a negative impact on preparing quality stained slides. Moreover, reutilization of microscope slides has been practicing. The chance of producing scratches on the surface of the slides during cleaning of the already used slides is very high. The scratches might be well stained during the staining steps and can confuse the observer and results to false positive test results [6]. This misleads the physician and the patients will be misdiagnosed and mistreated. This will not end up with over estimation of malaria in the region, economic loss for the patients and the nation, but also death of the individual with improper diagnosis and treatment. The unavailability of guideline and slide might not be due to either unavailability or shortage of supplies in the health institutes, but rather negligence of the responsible health care office in ordering guidelines and purchasing of slides.

Ninety percent of the respondents had not participated in in-service training regarding malaria microscopy in their work experience. The quality of accurate diagnosis of the parasite remains a challenge for most health institutions. Refresher training is thus important to ensure effective strategies that facilitate changes and adoption in the laboratory diagnosis of malaria. The importance of clear standard operating procedures, budgeting for the laboratory items and for training of the laboratory technicians separately and providing in-service training on laboratory diagnosis of malaria is important [9,11].

The gold standard for parasite detection is thick and thin blood smears, stained with Giemsa [5-8]. However, more than 50 percent of the technicians failed to use such gold standard technique in identification and quantification of malaria parasites. These showed the questionability of diagnostic tools in the study area. Moreover, 27.6% of them used thin blood smear only, which had low sensitivity compared with both thick and thin blood smear alone. Thus, there may be relatively high rate of false negativity. On the other hand, 7 (24.1%) of the technician used thick smear only. This again may result difficulty in correctly identifying the circulating malaria species in the patients [5,7]. In this survey, 72.4% of the respondents used binocular microscopes that used solar light as the source of illumination which could be one of the challenges in comprising the quality of microscopy services. Thus, providing high quality microscopes improves diagnostic accuracy in the malaria control and prevention system and guarantees the precise diagnosis and thereby proper malaria cases treatment in Ethiopia.

In this study, 79.3% of the study subjects reported that there was no quality control scheme done so far regarding blood films microscopy. This might be due to the absence of national blood smear microscopy quality control system. Though there is ‘Malaria and other Vector Borne Disease Department’ from national to District Health Office, quality control program on blood smear microscopy has not been practiced. This might be due to the lack of trained human power who able to perform quality control assessment on blood smear. We have observed the professional profile of the individuals working on Amhara Regional Sates ‘Malaria and other Vector Borne Disease Department’, and we couldn’t find a relevant professional who potentially offer malaria microscopy quality control operations. However, the office in collaboration with the Regional Health Research Laboratory should undertake a regular quality control program, so that problem associated with poor laboratory practice could have been identified. As per our understanding, there is no clear job description that should perform a regular quality control program on blood smear microscopy in the Regional Health Department. Thus, establishing a regional network of laboratory 'supervisors’ is crucial. To do so a team comprising senior technicians from each of Amhara administrative zones should be chosen by the Amhara Regional State Health Research Laboratory to constitute the regional network of laboratory supervisors who would implement quality control programme. They should be selected on the basis of their geographic location, seniority, technical qualifications, previous work experience and commitment to improving laboratory services. The study revealed that 96.6% of the study subjects had reported the species, stages and parasite density, which is the standard way of reporting. However, on the other hand, respondents failed to use the standard technique which was helpful for correct identification of the species of the parasites and to quantify the parasitic load. The mean number of blood smear slides examined per day in the institutions and the mean time to read single blood smear slide is in agreement with finding of similar study [10-12]. Overall, the findings of this study could be interpreted cautiously in lights of Amhara regional state since efforts to strengthen malaria microscopy diagnosis over the last few years by other Regional States (such as Oromia and Dire Dawa) in Ethiopia might come with different outcome.

## Conclusion

In conclusion, a large number of blood films continue to be handled, despite the poor technical procedures and lack of guidelines and logistics in the health-care laboratories. This calls for an urgent need to improve the laboratories by providing sufficient and appropriate working materials including high quality microscopes that are necessary for blood smear microscopy. In addition, there seems to a need for continued training and regular supervision of the staff, provision of guidelines and quality assessment tool for blood smear microscopy, which would increase the accuracy of malaria diagnosis. The Regional Health Bureau and the Regional Health Research Laboratory should harmonically work to promote improvement of the malaria microscopy in all health care facilitates and to conduct a regular quality control assessment by establishing a regional network of laboratory supervisors. Health managers need to secure funding for laboratory quality assurance programs at all levels. This includes establishing internal quality control measures and external validation systems that are linked into regional and national and if possible international external quality monitoring schemes, although implementing a quality assurance system for laboratories in developing countries is expensive and logistically complicated.
